# Characterization of the complete plastome of *Eleusine coracana* (Gramineae), an annual crop

**DOI:** 10.1080/23802359.2021.1899874

**Published:** 2021-03-18

**Authors:** Yuan Liu, Xin-Yan Yang, Yan Yao, Meng Zhang, Xue-Jie Zhang

**Affiliations:** a Shandong Provincial Key Laboratory of Plant Stress Research, College of Life Sciences, Shandong Normal University, Jinan, China; b Campus Hospital, Taishan University, Tai'an, China

**Keywords:** *Eleusine coracana*, plastome, phylogenomics

## Abstract

*Eleusine coracana* is a hardy crop that can grow in diverse environments. In this study, the complete plastome of *E. coracana* was determined. The plastome was 135,144 bp in size. It consists of a large single-copy region (80,666 bp), a small single-copy region (12,640 bp), and two inverted repeat regions (20,919 bp). The overall guanine-cytosine (GC) content was 38.2%. A total of 111 unique genes were annotated, including 77 protein-coding genes (PCGs), 30 tRNAs, and 4 rRNAs. Phylogenetic analysis showed that *Eleusine* was sister to *Dactyloctenium*.

*Eleusine coracana* belongs to Chloridoideae of Gramineae. As an annual cereal crop, *E. coracana* is originated in Africa and distributed in tropical and subtropical regions of the Eastern Hemisphere (Chandrashekar 2010). The species *E. coracana* can be cultivated from the Himalayas to coastal plains (Sood et al. 2019). *Eleusine coracana* has a wide range of uses. It can not only be used for food, forage and medicine, but also as green manure to improve soil condition (Kannan 2010; Devi et al. 2014; Sood et al. 2016; Divya et al. 2019). It has the characteristics of salt tolerance, barren tolerance, drought tolerance, and natural threshing (Rahman et al. 2014; Goron et al. 2015; Bartwal et al. 2016). In areas with harsh natural conditions, *E. coracana* can be introduced for cultivation to meet the demand for grain (Hittalmani et al. 2017). Therefore, obtaining genomic resources is of great significance for understanding and utilizing crops (Liu et al. 2020). In this study, we determined the plastome of *E. coracana*, which would provide basic genetic resources for studying this important species and solving its phylogenetic placement.

Fresh leaves of *E. coracana* were collected from Matou town, Tancheng county, Linyi city, Shandong province, China (34°39′ N, 118°18′ E). Voucher specimen (SD168) was deposited at College of Life Sciences, Shandong Normal University. Modified CTAB method was used to extract the total genomic DNA and sequenced using the Illumina Novaseq platform at Novogene (Beijing, China) (Wang et al. 2013). There are 19,704,688 reads generated for *E. coracana*. Plastome assembly was performed with Organelle Genome Assembler (OGA) (Qu, Fan, et al. 2019), with *E. indica* (NC_030486) as reference. The mean sequencing depth was 1185.5× for plastome of *E. coracana*. Annotation was performed with Plastid Genome Annotator (PGA) (Qu, Moore, et al. 2019). Geneious v9.1.4 was used for manual annotation correction. The annotated complete plastome was submitted to GenBank with the accession number MW262987. Alignment of 77 PCGs was conducted using MAFFT v7.3130 (Katoh and Standley 2013). A maximum likelihood (ML) tree was reconstructed using RAxML v8.2.10 (Stamatakis 2014), including tree robustness assessment using 1000 rapid bootstrap replicates with GTRGAMMA substitution model.

The complete plastome of *E. coracana* was 135,144 bp in size. It consists of a large single-copy region (80,666 bp), a small single-copy region (12,640 bp), and two inverted repeat regions (20,919 bp). The GC content was 38.2%. A total of 111 unique genes were annotated, including 77 PCGs, 30 tRNAs, and 4 rRNAs. Phylogenetic analysis showed that *Eleusine* was sister to *Dactyloctenium* (Figure 1).

**Figure 1.**
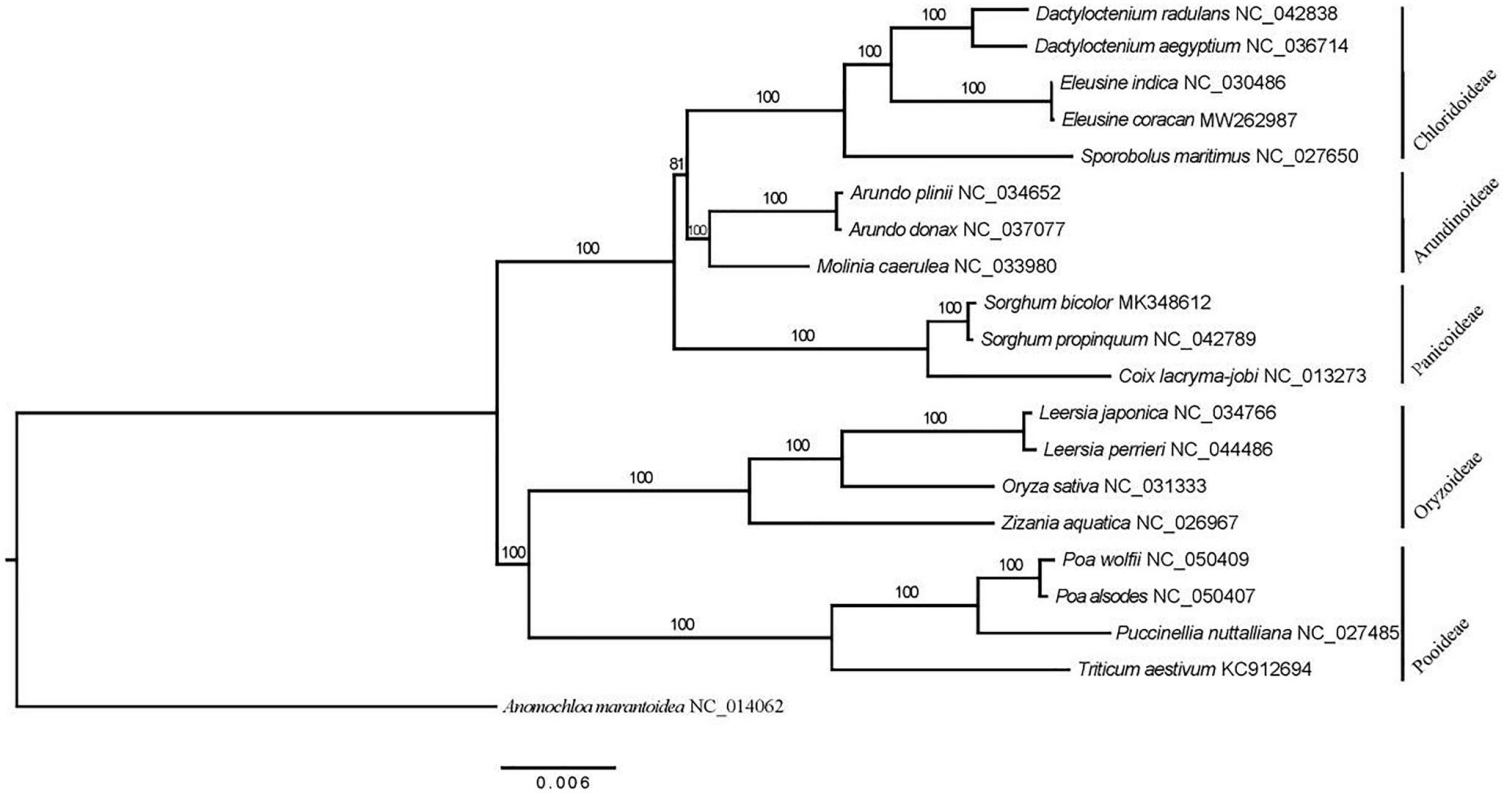
A maximum-likelihood (ML) tree inferred from 77 plastome genes. *Anomochloa marantoidea* from Anomochlooideae was used as outgroup. The numbers on branches are bootstrap support values.

## Data Availability

The genome sequence data that support the findings of this study are openly available in GenBank of NCBI at (https://www.ncbi.nlm.nih.gov/) under the accession no. MW262987. The associated BioProject, SRA, and Bio-Sample numbers are PRJNA678843, SRR13071189, and SAMN16815289, respectively.

## References

[CIT0001] Bartwal A, Pande A, Sharma P, Arora S. 2016. Intervarietal variations in various oxidative stress markers and antioxidant potential of *Finger millet* (*Eleusine coracana*) subjected to drought stress. J Environ Biol. 37(4):517–522. 27498495

[CIT0002] Chandrashekar A. 2010. *Finger millet*: *Eleusine coracana*. Adv Food Nutr Res. 59:215–262. 20610177 10.1016/S1043-4526(10)59006-5

[CIT0003] Devi PB, Vijayabharathi R, Sathyabama S, Malleshi NG, Priyadarisini VB. 2014. Health benefits of *Finger millet* (*Eleusine coracana* L.) polyphenols and dietary fiber: a review. J Food Sci Technol. 51(6):1021–1040. 24876635 10.1007/s13197-011-0584-9 PMC4033754

[CIT0004] Divya M, Sivashanmugam K, Ravi C, Govindarajan M, Alharbi N, Km S, Khaled J, Almanaa T, Vaseeharan B. 2019. Isolation of β-glucan from *Eleusine coracana* and its antibiofilm, antidiabetic, antioxidant, and biocompatible activities. Microb Pathog. 140(103955). 10.1016/j.micpath.2019.103955 31899325

[CIT0005] Goron TL, Bhosekar VK, Shearer CR, Watts S, Raizada MN. 2015. Whole plant acclimation responses by *Finger millet* to low nitrogen stress. Front Plant Sci. 6:652. 26347768 10.3389/fpls.2015.00652 PMC4541148

[CIT0006] Hittalmani S, Mahesh HB, Shirke MD, Biradar H, Uday G, Aruna YR, Lohithaswa HC, Mohanrao A. 2017. Genome and transcriptome sequence of *Finger millet* (*Eleusine coracana* (L.) Gaertn.) provides insights into drought tolerance and nutraceutical properties. BMC Genomics. 18(1):465. 28619070 10.1186/s12864-017-3850-z PMC5472924

[CIT0007] Kannan S. 2010. *Finger millet* in nutrition transition: an infant weaning food ingredient with chronic disease preventive potential. Br J Nutr. 104(12):1733–1734. 20673383 10.1017/S0007114510002989

[CIT0008] Katoh K, Standley DM. 2013. MAFFT multiple sequence alignment software version 7: improvements in performance and usability. Mol Biol Evol. 30(4):772–780. 23329690 10.1093/molbev/mst010 PMC3603318

[CIT0009] Liu Y, Zhang X, Han K, Li R, Xu G, Han Y, Cui F, Fan S, Seim I, Fan G, et al. 2020. Insights into amphicarpy from the compact genome of the legume *Amphicarpaea edgeworthii*. Plant Biotechnol J. 10.1111/pbi.13520 PMC8131047 33236503

[CIT0010] Qu X-J, Fan S-J, Wicke S, Yi T-S. 2019. Plastome reduction in the only parasitic gymnosperm parasitaxus is due to losses of photosynthesis but not housekeeping genes and apparently involves the secondary gain of a large inverted repeat. Genome Biol Evol. 11(10):2789–2796. 31504501 10.1093/gbe/evz187 PMC6786476

[CIT0011] Qu X-J, Moore M, Li D-Z, Yi T. 2019. PGA: a software package for rapid, accurate, and flexible batch annotation of plastomes. Plant Methods. 15(1):1–12. 31139240 10.1186/s13007-019-0435-7 PMC6528300

[CIT0012] Rahman H, Jagadeeshselvam N, Valarmathi R, Sachin B, Sasikala R, Senthil N, Sudhakar D, Robin S, Muthurajan R. 2014. Transcriptome analysis of salinity responsiveness in contrasting genotypes of *Finger millet* (*Eleusine coracana* L.) through RNA-sequencing. Plant Mol Biol. 85(4–5):485–503. 24838653 10.1007/s11103-014-0199-4

[CIT0013] Sood S, Joshi DC, Chandra AK, Kumar A. 2019. Phenomics and genomics of *Finger millet*: current status and future prospects. Planta. 250(3):731–751. 30968267 10.1007/s00425-019-03159-6

[CIT0014] Sood S, Kumar A, Babu BK, Gaur VS, Pandey D, Kant L, Pattnayak A. 2016. Gene discovery and advances in *Finger millet* [*Eleusine coracana* (L.) Gaertn.] genomics-an important nutri-cereal of future. Front Plant Sci. 7:1634. 27881984 10.3389/fpls.2016.01634 PMC5101212

[CIT0015] Stamatakis A. 2014. RAxML version 8: a tool for phylogenetic analysis and post-analysis of large phylogenies. Bioinformatics. 30(9):1312–1313. 24451623 10.1093/bioinformatics/btu033 PMC3998144

[CIT0016] Wang H-Y, Jiang D-F, Huang Y-H, Wang P-M, Li T. 2013. Study on the phylogeny of *Nephroma helveticum* and allied species. Mycotaxon. 125(1):263–275.

